# From malaria fighter to diabetes guardian: the emerging role of artesunate in treating diabetes and diabetic complications

**DOI:** 10.3389/fphar.2025.1583575

**Published:** 2025-05-22

**Authors:** Dongze Li, Qingyue Liang, Linghao Xu, Li Zhang, Qiming Gong, Tingting Zhou, Changfang Luo, Wei Huang, Yuan Yang

**Affiliations:** ^1^ Department of Endocrinology and Metabolism, The Affiliated Hospital of Southwest Medical University, Luzhou, Sichuan, China; ^2^ Sichuan Clinical Research Center for Nephropathy, Luzhou, Sichuan, China; ^3^ Metabolic Vascular Disease Key Laboratory of Sichuan Province, Luzhou, Sichuan, China; ^4^ Sichuan-Chongqing Joint Key Laboratory of Metabolic Vascular Diseases, Luzhou, Sichuan, China; ^5^ Department of Nutrition, Chengdu Seventh People’s Hospital, Chengdu, Sichuan, China; ^6^ Department of Du’s Orthopedic Surgery, Sichuan Second Hospital of Traditional Chinese Medicine, Chengdu, Sichuan, China; ^7^ Department of Neurology, The Affiliated Hospital of Southwest Medical University, Luzhou, Sichuan, China

**Keywords:** artesunate, artemisinin derivatives, malaria, diabetes mellitus, diabetic complications

## Abstract

Diabetes mellitus (DM) is a metabolic disease influenced by both genetic and environmental factors. The global incidence of DM is rising, and its multiple complications seriously affect patients’ quality of life and create a huge economic burden. At present, the prevention and treatment of DM mainly rely on oral or subcutaneous drugs, although oral drugs are more acceptable, they may produce more side effects and have limited effect on the treatment of diabetic complications. Artesunate (ART) is a first-line antimalarial drug widely used worldwide. Whether orally or intravenously, ART has high bioavailability and excellent pharmacokinetic properties in humans, and has shown good tolerance and safety in patients of multiple ages. Recent pharmacological studies have shown that, except for its antimalarial properties, ART also has a wide range of therapeutic potential for DM and its complications. This review aims to synthesize the latest research results, summarize and discuss the current role and mechanism of ART in improving diabetes and its complications, and provide a theoretical basis for the subsequent exploration of the anti-diabetes mechanism and the development of new antidiabetic agents based on ART, which has great clinical significance for strengthening the prevention and treatment effects of DM and its complications.

## 1 Introduction

Diabetes mellitus (DM) is a metabolic disorder arising from a combination of genetic and environmental factors, primarily characterized by either an absolute or relative deficiency in insulin secretion as well as decreased sensitivity to insulin action (Lu et al., 2024). The two most prevalent forms of DM are type 1 diabetes mellitus (T1DM) and type 2 diabetes mellitus (T2DM). According to the latest data released by the International Diabetes Federation (IDF), approximately 537 million people worldwide are living with DM as of 2021, and this figure is expected to rise to 783 million by 2045. In addition, all-cause mortality is 1.5–2 times higher in patients with DM than in those without DM (Kowalska et al., 2022). Acute and chronic complications related to DM are the main causes of disability and death in patients with DM. Acute complications include diabetic ketoacidosis, hyperglycemic hyperosmolar syndrome, and hypoglycemia. Chronic complications include microvascular diseases (e.g., diabetic retinopathy), macrovascular diseases (e.g., atherosclerosis), diabetic neuropathy, and diabetic foot. These complications seriously affect the quality of life of diabetic patients and bring huge economic burden to patients’ families and even the whole society. Consequently, it is of great significance to actively seek effective means to treat DM and its complications.

At present, the treatment of DM and its complications mainly relies on drug therapy, surgical treatment, and lifestyle interventions, among which oral hypoglycemic drugs are the most basic and most acceptable treatment. Traditional oral hypoglycemic drugs, including biguanides, sulfonylureas, and α-glucosidase inhibitors, may not only cause gastrointestinal reactions, weight gain, hypoglycemia risk, lactic acidosis, and other side effects (Kounatidis et al., 2024), but also have many therapeutic limitations. For example, they cannot directly act on the heart, liver, kidney, and other target organ damage caused by hyperglycemia, and the therapeutic effect varies greatly among individuals (Saturnino et al., 2025). Since DM involves a variety of metabolic disorders, its complications are numerous, and the underlying pathogenesis is complex, it is urgent to explore novel oral hypoglycemic drugs that can improve a variety of metabolic disorders, provide multi-organ protection, and have fewer side effects. In recent years, the research strategy of “new use of old drugs” has become a hot direction of drug development, and the time and capital investment of drug development can be greatly reduced by verifying whether the marketed drugs have the therapeutic potential of DM and its complications.

Artemisinin is a sesquiterpene lactone derived from the traditional Chinese medicine Artemisia apiacea, and has been used as a first-line treatment for malaria since its discovery in the 1970s (White and Chotivanich, 2024). Its discoverer, Tu Youyou, won the 2015 Nobel Prize in Physiology or Medicine for this breakthrough (Tiwari and Chaudhary, 2020). Artesunate (ART), also known as dihydroartemisinin-12-α-succinate, is a semi-synthetic derivative of artemisinin. Compared with artemisinin, whether administered orally or intravenously, ART has higher bioavailability, optimized pharmacokinetics and enhanced antimalarial therapeutic effect, and has demonstrated excellent human safety and tolerability. As a result, ART has become the most widely used antimalarial drug in clinical practice worldwide (Daily et al., 2022). It is worth noting that, beside its antimalarial properties, preclinical studies based on diabetes models published in the last 5 years have shown that ART not only lowers fasting blood glucose (FBG), improves glucose tolerance, and reverses insulin resistance (Chen et al., 2021; Wu et al., 2022a), but also has direct beneficial effects on a variety of diabetic target organ injuries (such as diabetic osteoporosis (Cen et al., 2023; Luo et al., 2024)). Moreover, ART can correct various metabolic disorders such as amino acid metabolism, bile acid metabolism, and glycerol phospholipid metabolism in DM (Guo et al., 2024; Chen et al., 2024).

This review aims to summarize the latest research evidence that ART improves DM and its complications by focusing on the underlying mechanisms. First, we briefly review the synthetic process of ART, the mechanism of antimalarial therapy, and metabolic processes in the human body. We then detail the beneficial effects of ART in T1DM and T2DM as well as its positive effects and regulatory mechanisms in improving multiple diabetic complications. This review provides a solid theoretical basis for promoting the multi-dimensional drug application of ART and has great clinical significance for promoting the understanding of the pathogenesis of DM and its complications and the development of novel oral drugs.

## 2 ART and its antimalaria effect

**FIGURE 1:**
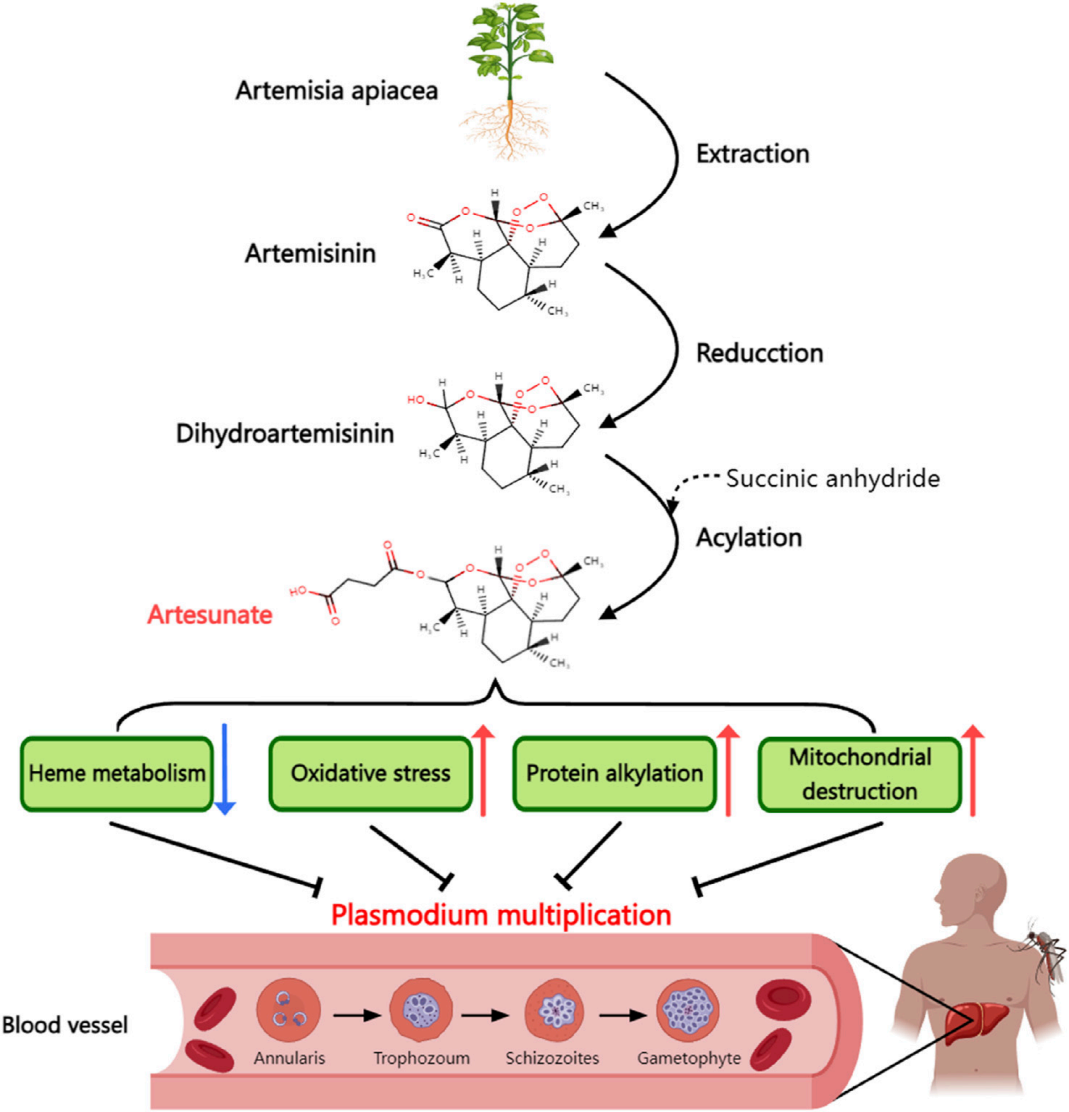
Synthetic process and antimalarial mechanism of artesunate. Artesunate is derived from artemisinin extracted from artemisinin in traditional Chinese medicine. Artemisinin is dehydrogenated and then acylated with succinic anhydride to form artesunate. Artesunate can inhibit the proliferation of Plasmodium in human body by inhibiting the heme metabolism of plasmodium, causing oxidative stress damage of plasmodium, alkylating plasmodium protein and directly causing mitochondrial dysfunction.

ART, with a molecular formula of C_19_H_28_O_8_ and molecular weight of 384.42 (Presser et al., 2017), is characterized as a white, odorless, relatively stable, and water-soluble crystalline powder. Its chemical production primarily occurs through esterification of dihydroartemisinin (DHA) with succinic anhydride (Figure 1).

The antimalarial mechanism of ART involves two crucial aspects. First, it binds to the microtubulin of Plasmodium, disrupting the microtubule arrangement and halting mitosis during the mid-stage, thus inhibiting its reproduction (Rocamora et al., 2018). Second, ART reacts with the Fe^2+^ produced during the decomposition of heme in the red blood cells of Plasmodium. This reaction leads to the destruction of the peroxy bridge in ART molecules and the release of numerous free radicals, rapidly killing Plasmodium cells (Combrinck et al., 2013). Because iron overload is associated with the development of T2DM, it is possible to lead to reduced insulin secretion and insulin resistance through mechanisms such as widespread oxidative stress and altered insulin signaling pathways (Simcox and McClain, 2013). In addition, the iron-chelating agent deferoxamine has been shown to improve liver insulin resistance by inhibiting ROS production (Wang et al., 2015). Therefore, ART may also alleviate iron overload through its iron-chelating properties, thereby improving T2DM, but there is currently no direct experimental evidence to prove the above hypothesis.

ART has demonstrated good safety and tolerability in humans, making it suitable for malaria patients of all ages, including both children and pregnant women. However, ART can still cause some side effects in rare cases, mainly gastrointestinal irritation such as nausea, vomiting (Rosenthal et al., 2024). ART metabolism primarily occurs in the liver, where it undergoes oxidation and reduction reactions mediated by liver cell enzymes. In the liver, ART is predominantly converted into its pharmacologically active metabolite, DHA. DHA is metabolized into various metabolites in the body, including monoamine oxides, carboxylic acids, and glucuronic acids (Kouakou et al., 2019). The metabolites of ART are primarily excreted via urine, with the excretion process potentially lasting for several days (Burger et al., 2016; Walker and Sullivan, 2017).

## 3 The effect of ART on DM

### 3.1 T1DM

T1DM is a pancreas-specific autoimmune disease characterized by CD4^+^ and CD8^+^ T cell-mediated destruction of β cells (Ilonen et al., 2019). Numerous clinical and experimental studies have demonstrated that ART exerts immunosuppressive effects across various autoimmune diseases, including bronchial asthma (Gu et al., 2023), systemic lupus erythematosus, multiple sclerosis, and inflammatory bowel disease (Gu et al., 2023; Gao et al., 2024). A recent study by Li et al. explored the immunomodulatory effects of ART in non-obese diabetic (NOD) mice, which can be used to simulate T1DM. Subsequently, the following phenomena were observed in the spleen, pancreas, and pancreatic lymph nodes: the proportion of CD4^+^ and CD8^+^ T cells producing the anti-inflammatory factor interleukin-4 (IL-4) increased significantly, whereas the number of T cells producing the pro-inflammatory factor interferon-γ (IFN-γ) decreased significantly. In addition, the mRNA levels of *tumor necrosis factor α* (*TNF-*α) and *interleukin-6* (*IL-6*) in the pancreas were reduced (Li et al., 2019). These findings suggest that ART alleviates T1DM primarily by regulating the balance between the subpopulations of T cells in the pancreas, that is, by reducing the number of autoimmune T cells and increasing the number of protective T cells, thereby preventing the destruction of the pancreas by the inflammatory response generated by the autoimmune cells. While this study advances our understanding of the role of ART in improving autoimmune diseases such as T1DM, it has certain limitations. Notably, it was not determined whether the increase in β cell count observed in NOD mice after ART administration was attributable solely to the immunosuppressive effects of ART, and it will be one of the future research directions to continue to investigate whether ART directly affects the differentiation and maturation of β cells.

In addition to immune regulation of the pancreas, T1DM is also associated with pyroptosis of β cells (Ding et al., 2019). Pyroptosis is a type of programmed cell death mediated by Csapase-1. Activated Caspase-1 can cut gasdermin D (GSDMD), thereby releasing the GSDMD-N fragment, which can insert into the cell membrane to form a hole, leading to cell rupture and the release of inflammatory factors such as interleukin-1β (IL-1β) and interleukin-18 (IL-18), leading to pyroptosis (Wang et al., 2025). Activation of the NOD-like receptor thermal protein domain associated protein 3 (NLRP3) inflammasome can lead to pyroptosis of β cells, exacerbate local inflammation of islets, and thus lead to insulin secretion disorder, which is one of the crucial pathological mechanisms of T1DM (Sun et al., 2020). Yuan et al. found that feeding ART to streptozocin (STZ) -induced T1DM mice reduced FBG levels, improved glucose tolerance, inhibited the expression of pancreatic Caspase-1 and GSDMD, and reduced serum IL-1β levels. They then demonstrated *in vitro* that this was caused by ART downregulating the expression of *NLRP3* in β-cells, thereby inhibiting STZ-induced pyroptosis of β cells (Yuan et al., 2022). These findings not only provide strong supporting evidence for the hypoglycemic effect of ART in T1DM but also reveal another potential mechanism by which ART improves T1DM over and above immune regulation. In the future, the effects of ART on other types of pancreatic cells besides β cells, such as α cells responsible for the secretion of glucagon and δ cells responsible for the secretion of somatostatin, should be investigated.

### 3.2 T2DM

ART not only improves T1DM but also alleviates T2DM by regulating glycolipid metabolism. A recent meta-analysis of 22 studies involving 526 animals (T2DM rats and db/db mice) comprehensively evaluated the efficacy of artemisinin derivatives, such as ART, in T2DM animal models. The overall results indicated that ART significantly reduced FBG levels, improved glucose tolerance and insulin sensitivity *in vivo*, and decreased HbA1c, total cholesterol (TC), triglycerides (TG), low-density lipoprotein (LDL), cholesterol, and free fatty acids (FFAs) (Wu et al., 2022b). This study suggests that ART may alleviate T2DM by enhancing glucose metabolism and improving lipid metabolism profile and insulin resistance. However, it is essential to note that most studies included in the meta-analysis had small sample sizes, which may have compromised the overall quality of evidence. This study also suggests that exploring how ART improves blood glucose and lipid metabolism disorders in patients with T2DM is a vital topic for future research. In the pathological course of T2DM, increased lipid deposition in β cells is strongly associated with decreased insulin sensitivity, elevated blood glucose levels, and impaired β cell function. Since ART has a broad spectrum of lipid-lowering effects, the study of the regulatory effect of ART intervention on lipid deposition in β cells is expected to open up new ideas for follow-up research in related fields.

Alagbonsi et al. investigated the effects of ART at different concentrations and durations of treatment on blood glucose regulation in normal rats of different sexes. In male rats, ART is effective in lowering blood glucose levels; however, long-term use of high-dose ART can cause elevated blood glucose levels. The researchers also observed that ART lowered blood glucose by promoting liver glycogen synthesis, inhibiting glucose-6-phosphate (G6P) enzyme activity, increasing plasma insulin concentration, and decreasing glucagon concentration. In addition, ART was found to reduce blood glucose in female rats only when administered at low doses for long periods, and ART did not affect plasma insulin levels in female rats (Alagbonsi et al., 2021). This reasearch reminds us that the effects of ART on the physiological regulation of blood glucose homeostasis are different under the conditions of different dosages and different duration, and the gender of the drug object is also an nonnegligible influencing factor. Consequently, it is necessary to further explore the effects of ART on various glucose-controlling hormones, such as insulin, glucagon, glucocorticoids, epinephrine, and thyroid hormones, in female and male bodies in T2DM.

A study published in 2024 used metabolomics, network pharmacology, and transcriptome sequencing analyses to comprehensively investigate the effects of ART on metabolic disorders in db/db mice and to elucidate the underlying mechanisms. The study found that ART not only significantly reduced the blood glucose and blood lipids of db/db mice and improved insulin resistance, but also significantly reduced the liver alanine aminotransferase (ALT), aspartate aminotransferase (AST), and oxidative stress indicators malondialdehyde (MDA) and superoxide dismutase (SOD) levels. In addition, ART-corrected disorders of glycerophospholipid metabolism, amino acid metabolism, cholic acid synthesis, and purine metabolism have been observed in the liver of db/db mice (Chen et al., 2024). Therefore, this not only confirms the results of the meta-analysis conducted by Abdullateef Isiaka Alagbonsi et al., but also suggests that ART may have a reverse effect on various metabolic disorders caused by oxidative stress in the liver of T2DM patients. In addition, liver transcriptome analysis and Western blotting were used to explore the mechanism of action of ART. On the one hand, ART upregulates the expression of glucose transporter 2 (Glut2) and insulin receptor substrate 1 (IRS1) proteins by promoting the phosphorylation of PI3K and AKT, and on the other hand reduces the phosphorylation of p38, ERK1/2 and JNK in MAPK signaling pathway (Table 1). These findings provide mechanistic evidence for the efficacy of ART in the treatment of T2DM. However, this study did not perform *in vitro* hepatocyte experiments or gene knockout/overexpression in the livers of animal models to further verify the role of the PI3K/Akt and MAPK pathways in ART improvement in T2DM. Another potentially fruitful avenue for future research is to compare the effects of ART on insulin resistance in the liver, skeletal muscle, and adipose tissue in T2DM models, and to explore other potential mechanisms besides the PI3K/Akt and MAPK pathways. Additionally, it is a promising research direction to compare the therapeutic effect of ART with metformin, GLP-1 receptor agonists, and other anti-T2DM drugs that have hypoglycemic effects and improve insulin resistance (Sirtori et al., 2024; Bednarz et al., 2022).

**TABLE 1:** Summary of studies on ART in the treatment of T1DM and T2DM.

Diseases	Year	Design	Model	Intervention	Outcome	Signal pathway	Ref.
T1DM	2019	*In vivo*	NOD mice	1.0 mg/mL ART, added in drinking water 9 weeks	ART reduced the expressions of pancreatic *TNF-*α and *IL-6* genes	Not identified	Li et al. (2019)
		*In vitro*	Primary mouse islet β cells	1 mM ART 24 h	ART increased the expressions of *Ins1*, *Ins2*, *MafA*, *Ucn3* and *NeuroD1* genes		
	2022	*In vivo*	C57BL/6J male mice were given a single intraperitoneal injection of 150 mg/kg STZ	0.5/1.0 mg/mL ART added in drinking water, 18 days	ART reduced fasting blood glucose and inhibited pancreatic pyroptosis	NLRP3 pathway	Yuan et al. (2022)
		*In vitro*	MINI6 cells were stimulated by STZ	0.8/1.6 μM ART 48 h	ART decreased The expression of NLRP3, Caspase-1 and GSDMD proteins		
T2DM	2024	*In vivo*	Male db/db mice	20/160 mg/kg/d ART, oral administration, 7 weeks	ART decreased the fasting blood glucose of db/db mice, improved insulin resistance and blood lipid profile, decreased serum MDA and SOD levels, increased the expression of pancreatic Glut2 and IRS1 proteins	PI3K/AKT and MAPK pathways	Chen et al. (2024)

Abbreviations: T1DM, type 1 diabetes mellitus; T2DM, type 2 diabetes mellitus; ART, artesunate; HFD, High-fat diet; STZ, streptozotocin; NOD, non-obese diabetes.

Progressive decline in islet β cell mass is one of the core pathological features of T2DM. In T2DM, the long-term insulin resistance of the body will promote the synthesis of a large number of insulin precursors by β cells, resulting in excessive accumulation of misfolded proteins in the endoplasmic reticulum, thereby inducing ER (endoplasmic reticulum) stress and resulting in loss of β cells. In addition, chronic hyperglycemia also leads to the generation of excessive reactive oxygen species by acting on the mitochondrial respiratory chain of β cells, causing the apoptosis of β cells and oxidative stress damage, leading to the decline of β cell quality. Therefore, ART may reverse the quality decline of islet β cells in T2DM by inhibiting the expression of pancreatic proapoptotic proteins such as Caspase-1 and regulating glucose metabolism homeostasis (Yuan et al., 2022; Wu et al., 2022b; Alagbonsi et al., 2021).

## 4 The effect of ART on diabetic complications

### 4.1 Diabetic osteoporosis

Diabetic osteoporosis (DOP) is a metabolic bone disease that is caused by DM. DOP is characterized by a decrease in bone mineral density, destruction of the bone microstructure, and increased bone fragility, leading to an increased risk of fracture. The effects of DM on bone metabolism are mainly reflected in the decrease in bone formation mediated by osteoblasts and increase in bone resorption mediated by osteoclasts (Ullah et al., 2024). Previous studies have investigated the effects and mechanisms of action of ART on bone formation and resorption. For instance, ART has been shown to inhibit LPS-induced osteoclast generation in RAW264.7 cells and to reduce LPS-induced bone loss in mouse femurs. Moreover, ART inhibits receptor activator of nuclear factor-κB ligand (RANKL)-induced osteoclast generation in primary bone marrow-derived macrophages by attenuating IκB degradation and NF-κB phosphorylation (Wei et al., 2018). Additionally, ART can suppress RANKL-induced osteoclast formation and bone resorption by inhibiting the PLCγ1-Ca^2+^-NFATc1 signaling pathway, and can reverse bone loss caused by ovariectomy by reducing *RANKL* expression levels (Zeng et al., 2017; Zeng XZ. et al., 2020). ART also ameliorates osteoporosis and inflammation following ovariectomy by inhibiting the expression of *NF-κB* and proteins associated with the Notch1/Hes1 signaling pathway (Wang et al., 2024). Accordingly, ART may be a promising option for the treatment of diabetic inflammatory bone loss. However, the aforementioned studies used LPS- or RANKL-induced osteoporosis models, which may more accurately simulate bone loss induced by infection or castration. Consequently, further *in vitro* and *in vivo* experiments in DM models are required to verify the effect of ART on DOP.

ART can also influence the occurrence and development of osteoporosis through miRNA regulation. Specifically, ART has been shown to inhibit the activity of osteoclasts derived from RAW264.7 cells by increasing the expression level of miR-503. miR-503 directly downregulates *RANKL*, which subsequently affects the downstream activation of the MAPK and AKT signaling pathways associated with RANKL, thereby reducing the activity and function of osteoclasts (Huang et al., 2020). Additionally, ART can induce the expression of miR-34a while downregulating the expression of DKK1, a known inhibitor of the Wnt signaling pathway. This action blocks the activity of the Wnt signaling pathway, ultimately promoting the differentiation of human bone marrow mesenchymal stem cells (MSCs) into osteoblasts (Zeng HB. et al., 2020). The above studies all indicated that the effect of ART on miRNA was dose-dependent. However, the effects of different ART concentrations on bone formation or bone resorption were not discussed, and the above two studies did not use miRNA inhibitors or mimics and luciferase reporter gene detection to further verify the regulatory relationship between miRNA and target genes. Through high-throughput RNA sequencing and mining of the Pharmaco-miR database, more miRNA targets of ART may be screened, and more abundant data will be provided for subsequent studies on the mechanism of ART treatment of osteoporosis.

A study published in 2024 demonstrated the therapeutic potential of ART in DOP. The researchers induced T2DM rats with HFD and STZ and subsequently used an ART-loaded thermosensitive chitosan hydrogel (ART-loaded TCH) to control the timing and dose of ART release. Different concentrations of ART-loaded TCH were then administered to study the effect of ART on alveolar bone mineral density in T2DM rats after tooth extraction (Luo et al., 2024). The results showed that ART significantly improved alveolar bone height and density, alleviated alveolar bone inflammation, promoted regeneration, and increased the expression of osteoprotectin (OPG) and alkaline phosphatase (ALP). In addition, the treatment group showed significant regulation of Th1 and Th2 cell release while inhibiting the expression of *MAPK* and *ERK1* in the upper jaw of T2DM rats (Table 2). Thus, ART may be an effective strategy for preventing DOP through its effects on improving bone inflammation, increasing bone density, and immune regulation. In addition, network pharmacology and molecular docking studies conducted by Ma et al. predicted that not only MAPK but also serine protease receptors, AMP-activated protein kinase (AMPK), prolactin, and prolactin signaling pathways may be potential mechanisms for ART to improve DOP (Ma et al., 2022). Future studies should focus on exploring and validating the efficacy of oral ART in the treatment of DOP and the role of the aforementioned highly correlated signaling pathways screened by bioanalysis in these therapeutic effects.

**TABLE 2:** Summary of studies on ART in the treatment of diabetic microvascular complications, diabetic osteoporosis, diabetic peripheral neuropathy and diabetic xerostomia.

Diseases	Year	Design	Model	Intervention	Outcome	Pathway	Ref.
Diabetic kidney disease	2018	*In vitro*	HBZY-1 cells stimulated by 30 mM glucose	5/30 μg/mL ART 24 h	ART inhibited cell proliferation, reduced inflammatory factors, ECM, ROS and MDA levels	TLR4/NF-κB/NLRP3 pathway	Sun et al. (2018)
Diabetic retinopathy	2021	*In vivo*	Male SD rats induced by STZ	2/10 mg/mL ART, intravitreal injection, once at the beginning of the experiment and once at the fourth week	ART inhibited the increase of retinal thickness, leukocyte adhesion, microglia activation, inflammatory cytokines and ROS production, and promoted the expression of Beclin-1 and the LC3II/I ratio in the retina	AMPK/SIRT1 pathway	Li et al. (2023)
Diabetic cardiomyopathy	2021	*In vivo*	Male SD rats induced by STZ	50/100 mg/kg/d ART, gavage, 4 weeks	ART decreased FBG and blood lipids, and decreased the expression of cardiac NF-κB, CD68, MMP1, MMP9 and RAGE	RAGE/NF-κB pathway	Chen et al. (2021)
Diabetic osteoporosis	2024	*In vivo*	Male SD rats induced by STZ + HFD	50/100/150 mg/mL ART dissolved in TCH, continuous external application	ART improved maxillary bone mineral density, promoted the expression of OPG, ALP and RANK, inhibited the release of Th1 and Th2 cells	MAPK pathway	Luo et al. (2024)
Diabetic peripheral neuropathy	2023	*In vivo*	Male SD rats induced by HFD + STZ	0.7 mg/mL ART, added to drinking water, 12 weeks	ART inhibited the apoptosis of sciatic nerve cells and improved the nerve injury	PI3K/AKT pathway	Zhang et al. (2023)
		*In vitro*	RSC96 cells stimulated by 25 mM glucose	0.15/0.3 μg/mL ART 48 h	ART inhibited the apoptosis of RSC96 cells		
Diabetic xerostomia	2021	*In vivo*	Male SD rats induced by HFD + STZ	50 mg/kg/d ART, tube feeding, 4 weeks	ART reduced urine volume and water intake, reduced FBG and blood lipid, improved glucose tolerance, improved salivary gland fibrosis and acinous cell mitochondrial damage, and promoted aquaporin AQP5 expression	PI3K/AKT pathway	Zhang et al. (2021)

Abbreviations: T1DM, type 1 diabetes mellitus; T2DM, type 2 diabetes mellitus; ART, artesunate; HFD, High-fat diet; STZ, streptozotocin; TCH, thermostatic chitosan hydrogel; FBG, fasting blood glucose.

### 4.2 Diabetic kidney disease

Hyperglycemia is the primary cause of diabetic kidney disease (DKD), which directly stimulates excessive proliferation and hypertrophy of mesangial cells and upregulates the expression of pro-inflammatory and pro-fibrotic genes, such as *NF-κB* and *TGF-β* (Młynarska et al., 2024). This excessive proliferation of mesangial cells results in increased secretion of the extracellular matrix (ECM), leading to thickening of the mesangial matrix and basement membrane, ultimately culminating in glomerular sclerosis (Jin et al., 2023). An *in vitro* experiment preliminarily evaluated the effect of ART on renal inflammation and ECM aggregation in DKD and its underlying molecular mechanism. The findings indicated that ART treatment inhibited the proliferation of HBZY-1 cells (rat mesangial cell line) induced by high glucose, and reduced the production of ECM and inflammatory factors such as IL-6, IL-1β, and TNF-α. Furthermore, ART decreased the levels of reactive oxygen species (ROS) and MDA in high-glucose-induced HBZY-1 cells and inhibited SOD activity. The protective effects of ART on DKD are believed to be associated with its inhibition of the TLR4/NF-κB/NLRP3 inflammasome pathway (Sun et al., 2018). This study suggests that ART may be used as an anti-inflammatory, anti-oxidative, and anti-fibrotic DKD therapy. Although this study provides preliminary evidence for ART as a potential treatment for DKD, it was limited to *in vitro* experiments. Further studies are warranted to validate its role in animal models of DKD and to explore the biological effects of ART on other mechanisms underlying the onset and progression of DKD.

A recently published meta-analysis reviewed the existing literature to systematically evaluate the efficacy and potential mechanisms of artemisinin and its derivatives in DKD treatment. A total of 18 articles were included, including 418 animal models, six of which used ART. The results showed that ART and other artemisinin derivatives reversed renal pathological changes and improved renal function indicators related to DKD, such as serum creatinine (SCr), blood urea nitrogen (BUN), and urinary albumin (UA) (Feng et al., 2022). The potential mechanism of action of these drugs may be closely related to delayed renal fibrosis, anti-inflammatory effects, relieving antioxidant stress, and enhancing renal autophagy. This conclusion is similar to the previously mentioned results of *in vitro* experiments on high-glucose-induced HBZY-1 cells (Sun et al., 2018). However, this meta-analysis also had some limitations, such as high heterogeneity in the results of BUN, SCr, and UA in some experiments and insufficient sample size for some indicators. In addition, all preclinical studies were conducted using male animals in the experimental design. So as that, there is an urgent need to conduct higher-quality preclinical trials involving both women and men to further validate and compare the efficacy of artemisinin derivatives such as ART in the treatment of DKD.

### 4.3 Diabetic periphera neuropathy

Diabetic peripheral neuropathy (DPN) has multiple clinical manifestations, including paresthesia, dyskinesia, and autonomic dysfunction, which seriously affects the quality of life of diabetic patients (Savelieff et al., 2024). The occurrence and development of DPN are related to the damage of microvessels supplying essential nutrients to nerves, an increase in inflammatory cells, and abnormal cytokine expression in peripheral nerves (Hagen and Ousman, 2021). Currently, specific drugs for DPN are lacking in clinical practice, and existing treatment strategies are mainly aimed at relieving symptoms and slowing disease progression, such as the use of painkillers, antioxidant stress agents, and neuronutrients (Baicus et al., 2024).

A recent study explored the potential of ART in preventing DPN. Researchers established DPN mice using HFD and STZ injections and found that ART improved performance on tests of pain behavior in DPN mice, including hot plate response and cold sensitivity. The results of H&E staining and immunofluorescence of the sciatic nerve showed that nerve injury was significantly reduced in the ART treatment group, which was manifested as a reduction in axon number and widening of nerve space. *In vitro* experiments further confirmed that ART improved the survival rate of RSC96 cells (rat nerve cell line) under high glucose conditions, decreased the expression of the pro-apoptotic protein Bax, and promoted the expression of the anti-apoptotic protein Bcl-2. Beyond that, the authors found that ART improved DPN by activating the PI3K/AKT/mTOR signaling pathway (Zhang et al., 2023). Thus, the results of this study provide new insights into drug treatment of DPN. In the study of ART and DOP conducted by Luo et al., a thermosensitive chitosan hydrogel containing ART was adopted (Luo et al., 2024). Accordingly, this special dosage form of ART that can be applied locally may play a significant role in the treatment of DPN. Further studies are needed to determine the optimal drug dosage and human safety of this particular dosage form for DPN treatment. In addition, it is crucial to explore the impact of ART on autonomic nervous dysfunction in cardiovascular, urinary, digestive, and other systems caused by diabetes.

### 4.4 Diabetic retinopathy

Diabetic retinopathy (DR) poses a significant threat to the visual health of diabetic patients, but current pharmacological treatments do not provide a complete cure. Li et al. investigated the effects of ART on STZ-induced DR in rats and explored the underlying mechanisms. Researchers discovered that intraretinal injection of ART inhibited the increase in retinal thickness in DR mice, reduced the number of FITC-coupled WGA lectin-labeled retinal white blood cells, and decreased the number of Iba1 immunolabeled microglia. ART treatment lowered the expression levels of *IL-1*β, *IL-6*, and *MCP-1* in the retina. They also observed that ROS production was elevated in the outer nuclear, inner core, and ganglion cell layers of the retina in DR rats, and ART significantly reduced ROS levels in these regions. Additionally, ART enhances retinal autophagy by promoting Beclin-1 expression and the LC3II/I ratio (Li et al., 2023). Consequently, ART may reverse the progression of DR through its anti-inflammatory, anti-oxidative stress, and autophagy-promoting effects, which further deepens our understanding of the anti-diabetic complications of ART. This study innovatively employed ART intraretinal injection to directly target the lesion site, thereby circumventing the absorption and metabolism issues associated with oral medications and enhancing therapeutic efficacy. On that account, ART may serve as an effective localized treatment for improving DR. However, this study did not include *in vitro* experiments or reverse experiments to further elucidate the mechanisms by which ART improves DR. Future research should continue to investigate the pharmacodynamic characteristics and broader pharmacological mechanisms of ART local administration in the retina.

### 4.5 Diabetic cardiomyopathy

Diabetic cardiomyopathy (DCM), one of the leading causes of death in patients with DM, is a structural and functional abnormality of the heart that is independent of hypertension, coronary atherosclerotic heart disease, and other cardiovascular diseases. The disturbance of metabolism of energy substrates, such as glucose, in cardiomyocytes can lead to a progressive decline in DCM cardiac function, myocardial fibrosis, and ventricular remodeling, eventually leading to heart failure (Ramachandra et al., 2021).

Chen et al. initially investigated the effect of ART on cardiac fibrosis in T1DM rats. They found that ART administration for 4 weeks in T1DM rats not only reduced FBG and blood lipids, but also inhibited the expression of pro-fibrotic factors MMP1 and MMP9 in the heart through immunohistochemical and qPCR tests. Further studies showed that the beneficial effect of ART on DCM hearts was due to the inhibition of the RAGE/NF-κB signaling pathway in the heart tissue (Chen et al., 2021). Unfortunately, the researchers did not evaluate whether ART improved the pumping function of the DCM heart and the structural abnormalities of the heart cavity using cardiac ultrasound. Nevertheless, the limited experimental results of this study suggest that ART may reverse DCM progression through its anti-myocardial fibrosis effect. Future studies should further investigate the mechanisms by which ART improves myocardial fibrosis in DCM as well as the effects of ART on cardiac insulin resistance, energy substrate metabolic perturbations, and mitochondrial dysfunction in DCM.

### 4.6 Diabetic xerostomia

Hyperglycemia leads to the excretion of excess glucose through the urine in patients with DM, which in turn causes significant water loss and may result in symptoms of dry mouth. Furthermore, hyperglycemia stimulates the thirst center in the hypothalamus, thereby increasing the body’s demand for water. Additionally, damage to salivary gland function caused by hyperglycemia is a significant contributor to the development of diabetic xerostomia (Rohani, 2019). Studies have demonstrated that metformin can ameliorate histological changes in the salivary glands of diabetic rats by reducing the expression of *TNF-*α and *IL-6* genes, which subsequently alleviates dry mouth symptoms and may offer protective effects on salivary gland function in diabetic patients (Kim et al., 2021). Moreover, ART alleviated the structural changes and dysfunction of lacrimal glands in diabetic rats by reducing the expression of *NF-κB* and *TNF-*α (Rajbanshi et al., 2024), suggesting that ART may have a similar effect as metformin in improving salivary gland dysfunction caused by inflammation.

The work of Siqin Zhang et al. compared the effects of metformin and ART in diabetic xerostomia. The results showed that ART alone and ART in combination with metformin significantly reduced blood glucose levels and improved various other phenotypes in T2DM rats, including weight loss, increased urine volume, increased water intake, insulin resistance, and abnormal glucose tolerance. In addition, ART showed the ability improved salivary gland damage. Interestingly, ART and metformin have similar therapeutic effects on inhibiting damage to salivary gland cells, the combination of ART and metformin was more effective than monotherapy. Further mechanistic studies showed that ART alleviated diabetic dry mouth syndrome by activating the PI3K/AKT signaling pathway to inhibit apoptosis and autophagy in salivary gland cells (Zhang et al., 2021). Based on this study, we can accelerate the development of ARt-based anti-diabetic dry mouth drugs. However, further studies are needed to determine whether ART also plays a role in parasympathetic innervation of salivary secretion.

### 4.7 Atherosclerosis

Vascular endothelial cells play a central role in atherosclerosis (AS). They attract monocytes to migrate to the intima of the blood vessel wall through the expression of adhesion molecules, such as VCAM-1 and ICAM-1 (Choi et al., 2022). These monocytes are subsequently transformed into macrophages that phagocytose oxidized low-density lipoproteins in the intima, forming foam cells, which are primary contributors to atherosclerotic plaque formation (Deng et al., 2023). Moreover, injury and dysfunction of endothelial cells are closely related to AS. For instance, activation of the NLRP3 inflammasome can induce pyroptosis in endothelial cells, resulting in the release of numerous inflammatory factors including IL-1β and IL-18. This process exacerbates the inflammatory response within the blood vessel wall and accelerates AS progression (Weber et al., 2023). For that reason, AS progression can be effectively mitigated or even reversed through strategies aimed at lowering blood lipid levels and reducing arterial inflammation.

Previous studies have confirmed the anti-inflammatory effects of ART in AS patients. Weiwei Jiang et al. demonstrated that ART can decrease the expression of IL-18 and IL-6 in the aorta of ApoE^−/−^ mice fed a western diet, thereby slowing the progression of AS in these animals. Furthermore, a study revealed that the combination of ART and rosuvastatin is more effective in improving AS than ART alone (Jiang et al., 2016), indicating that a synergistic approach utilizing both anti-inflammatory and lipid-regulating medications may offer greater benefits for patients with AS. More importantly, ART showed broad-spectrum lipid-lowering effects comparable to those of atorvastatin in db/db mice (Cen et al., 2023).

In summary, ART shows obvious anti-arterial inflammation and local lipid deposition in the blood vessels in the treatment of AS (Table 3). However, current studies on ART and AS are mainly conducted on non-diabetic models, so the anti-AS effects of ART urgently need to be further verified on *in vivo* and *in vitro* diabetes models. Future studies should continue to explore whether ART affects the absorption and utilization of lipids from food sources and its influence on other lipid metabolism regulatory enzymes besides LPL. From another perspective, we can also study the combined effects, interactions, and pharmacokinetic effects of ART and other anti-AS drugs, such as atorvastatin, in order to find a more effective treatment plan for AS.

**TABLE 3:** Summary of studies on ART in the treatment of diabetic macrovascular complications.

Disease	Year	Design	Model	Intervention	Outcome	Pathway	Ref.
Atherosclerosis	2021	*In vitro*	PMA-induced THP1 cells	1.5/5/15 μg/mL ART 12 h	ART increased the expression of ABCA1 and ABCG1 by inhibiting TLR4, reduced the ox-LDL-induced cell TNF-α and IL-6 secretion	TLR4/ABCA1/ABCG1 pathway	Qian et al. (2021)
	2022	*In vivo*	Male ApoE^−/−^ mice + HFD	60 mg/kg/d ART, tube feed, 8 weeks	ART inhibited the polarization of macrophages into M1-like phenotype, and improved lipid, amino acid and bile acid metabolism disorders	HIF-1α and NF-κB pathways	Wang et al. (2022)
		*In vitro*	LPS-stimulated RAW 264.7 cells and mouse primary macrophages	10/20 μM ART 24 h	ART decreased the expression of HIF-1α and inhibited the phosphorylation of NF-κB and IκBα		
	2023	*In vivo*	Male Wistar rats were induced by HFD + VD3 intradurally	1.5/4.5 mg/kg/day ART, Added to HFD 8 weeks	ART downregulated the expression of NLRP3, ASC, Caspase-1, IL-1β, IL-18, TGF-β1 in aorta	NLRP3 pathway	Cen et al. (2023)
	2024	*In vivo*	Male ApoE^−/−^ mice + HFD	10 mg/kg/d ART, intraperitoneal injection, 12 weeks	ART decreased the expression of NLRP3, CD68, IL-1β, MMP9 and KLF4	ERK1/2/NF-κB/NLRP3 pathway	Liu et al. (2024)
		*In vitro*	LPS + TNF-α stimulated HVSMCs cells	Not identified	ART decreased the expression of NLRP3, Caspase-1 and IL− 1β		
Myocardial infarction	2018	*In vivo*	Male Wistar rats after closure of left anterior descending coronary artery	1 mg/kg ART, once for cardiac reperfusion	ART reduced myocardial infarction area, promoted phosphorylation of cardiac Akt and eNOS, inhibited nuclear translocation of NF-κB	PI3K/Akt/NF-κB/NLRP3 pathway	Khan et al. (2018)

Abbreviations: ART, artesunate; VD3, Vitamin D3; LPS, lipopolysaccharide; HFD, High-fat diet; CHD, coronary atherosclerotic heart disease; PMA, phorbol myristate acetate; HUVECs, human umbilical vein endothelial cells; VSMCs, Vascular smooth muscle cells; ox-LDL, oxidized low-density lipoprotein; HVSMCs, human vascular smooth muscle cells; α-SMC, α-smooth muscle actin.

### 4.8 Myocardial infarction

Coronary atherosclerosis caused by DM may cause platelets to accumulate in the coronary endothelium, forming blood clots that narrow or block the coronary arteries, thereby reducing the blood supply to the heart and causing myocardial infarction (MI) (Zhu et al., 2024). In addition, hyperlipidemia increases the risk of MI in individuals with DM (Sosnowska et al., 2024). As ART has a broad spectrum of lipid-lowering effects (Sun et al., 2020), it is of great clinical value to explore whether it can reduce the occurrence of MI.

A previous study in cardiac ischemia-reperfusion (I/R) rats hinted at the potential effects and mechanisms of ART in reducing MI size. The researchers first closed the left anterior descending coronary artery of rats for 2 h, and then administered ART intravenous injection of ART during cardiac reperfusion. It has been found that ART may ameliorate cardiac inflammation and oxidative stress injury after I/R through its inhibitory effect on Akt phosphorylation in cardiac tissue (Khan et al., 2018). In addition, a recent study based on platelets sourced from healthy volunteers explored the effects of ART on platelet aggregation and thrombosis. The results showed that ART significantly increased the production of cyclic adenosine phosphate (cAMP) and cyclic guanosine phosphate (cGMP) in platelets. Moreover, ART significantly inhibits phosphorylation of MAPK and the PI3K/Akt pathway in platelets, resulting in decreased production of thromboxane A2 (TXA2) and decreased release of intracellular platelet granular secretions, such as 5-hydroxytryptamine (5-HT) and adenosine triphosphate (ATP) (Yoon et al., 2022). Consequently, ART has the effect of anti-platelet aggregation and thrombosis and is expected to become a new drug for the prevention and treatment of cardiovascular diseases such as MI. However, it should be noted that this study was conducted *in vitro*, and the experimental conditions may not fully replicate the physiological and pathological state of the human body. Consequently, the actual effect of ART on anti-platelet aggregation requires further validation through clinical trials. It cannot be ignored that the risk of bleeding and other potential side effects of long-term use of ART need to be thoroughly evaluated. Other studies have shown that ART can significantly increase the expression level of IRF4, promote the polarization of M2-type macrophages, and inhibit the polarization of M1-type macrophages, thus reducing the degree of vascular stenosis. Therefore, this could be another potential mechanism for ART to improve MI (Miao et al., 2025).

More importantly, Since most of the existing studies on ART and MI focus on non-diabetic animal models, the effectiveness of their antiplatelet effects in diabetic conditions has not been fully demonstrated. Therefore, it is urgent to validate and evaluate the anti-platelet aggregation effect of ART in diabetic models.

## 5 Conclusions and prospects

**FIGURE 2:**
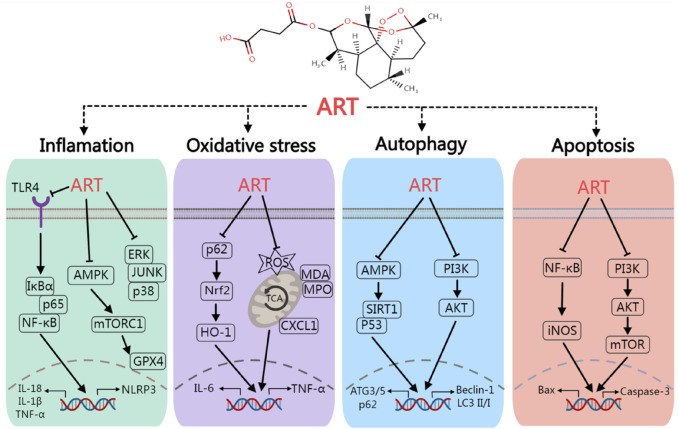
The mechanism of artesunate regulate inflammation, oxidative stress, autophagy and apoptosis. Artesunate can ameliorate diabetes mellitus and its chronic complications by regulating various signaling pathways such as TLR4/NF-κB, AMPK/SIRT1 and PI3K/AKT.

**FIGURE 3:**
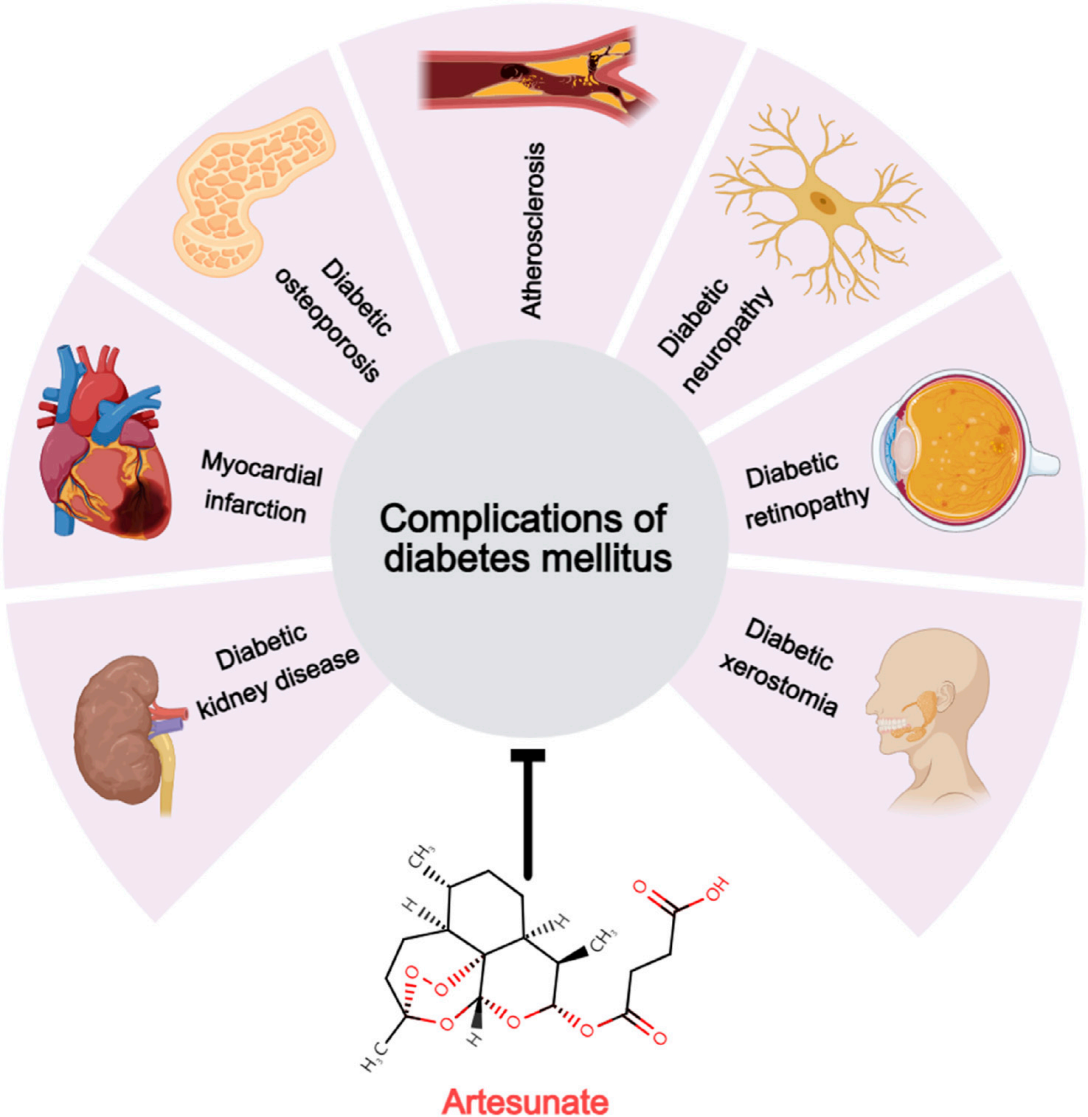
Therapeutic potential of artesunate for diabetic complications. Artesunate has a therapeutic effect on the development and development of multiple chronic diabetic complications, including diabetic kidney disease, myocardial infarction, diabetic osteoporosis, atherosclerosis, diabetic neuropathy, diabetic retinopathy, and diabetic dry mouth disease.

Numerous preclinical studies have demonstrated that ART, a widely used antimalarial drug known for its excellent tolerance and minimal side effects, can mediate various signaling pathways that are unrelated to its antimalarial mechanism (Figure 2), including the PI3K/Akt (Chen et al., 2024), MAPK (Yoon et al., 2022), NF-κB/RANKL (Wei et al., 2018), PLCγ1-Ca^2+^-NFATc1 (Zeng et al., 2017), and Notch1/Hes1 pathways (Wang et al., 2024). Through these mechanisms, ART influences a range of pathophysiological processes such as oxidative stress (Chen et al., 2024), immune response (Li et al., 2019), pyroptosis (Yuan et al., 2022), glycolipid metabolism (Wu et al., 2022b), and autophagy (Li et al., 2023). Consequently, ART has exhibited a broad spectrum of beneficial effects in the treatment of T1DM, T2DM, and various chronic complications associated with DM (Figure 3),based on its multi-target effect, ART may become an adjunctive therapeutic drug based on traditional antidiabetic drugs such as metformin and insulin.

Although existing studies have demonstrated the pharmacological value of ART in managing DM and its complications, relevant research is still in its nascent stages. Many limitations in the experimental design of current studies significantly undermine the overall credibility of the evidence. For instance, most investigations primarily focused on the short-term effects of ART, neglecting a comprehensive discussion of the long-term safety and efficacy of the medication. Additionally, some studies have utilized ART doses that are excessively high and may exceed those typically employed in the clinical treatment of human malaria. Furthermore, the absence of relevant clinical trials raises concerns as the effects and mechanisms observed in animal and cell models may not fully align with those in human subjects. In many of these studies, the underlying mechanisms of action associated with ART have not been thoroughly investigated. Some studies lack corroborative evidence from both *in vivo* and *in vitro* experiments, while others fail to incorporate exclusive and reversal experiments to explore the mechanisms involved. Accordingly, it is imperative that future studies address these limitations and further delineate research directions that warrant exploration based on the current state of knowledge.

Current studies primarily confirm the role of ART in chronic complications of DM. Furthermore, the role and potential mechanisms of ART in acute complications such as diabetic ketoacidosis, hyperosmolar hyperglycemic coma, and hypoglycemic reactions warrant further investigation. Additionally, exploring the combined hypoglycemic effects of ART with other antidiabetic medications, excluding metformin, as well as the pharmacokinetic interactions between these drugs, holds significant clinical importance for future studies. Investigating the feasibility of developing ART-based composite preparations is recommended. Given that intravenous injection is the most effective method of administering ART, it is essential to assess whether this route can enhance DOP or DKD. Moreover, evaluating the potential of thrombolytic therapy for MI via interventional surgery and the innovative delivery of ART to poorly perfused organs, such as the heart and brain, using nanoparticle drug delivery systems represents a promising research avenue. Further research into the effects of oral administration of ART on DM and its complications, particularly regarding its influence on the gut microbiota and specific metabolites, will deepen our understanding of its pharmacological mechanisms. Since ART is derived from succinic anhydride acylation of DHA, it is crucial to examine whether ART can alleviate diabetic complications by promoting succinylation modifications. Finally, continued exploration of the differential effects of ART on glucose and lipid metabolism in diabetic patients across various ages and sexes will contribute meaningfully to the field of DM research and its complications.

In conclusion, as drug research continues to develop, we will deepen our understanding of the pharmacological mechanisms of ART. We anticipate that future studies will address these current limitations, accelerate the translation of basic research into clinical practice, and provide a stronger theoretical foundation for the development and practical application of ART-based adjuvant drugs for diabetes and its complications.

## Abbreviations

DM, diabetes mellitus; T1DM, type 1 diabetes mellitus; T2DM, type 2 diabetes mellitus; ART, artesunate; DHA, dihydroartemisinin; NOD, non-obese diabetes; IL-4, interleukin-4; IL-6, interleukin-6; IL-18, interleukin-18; IL-1β, interleukin-1β; IFN-γ, interferon-γ; TNF-α, tumor necrosis factor-α; NLRP3, NOD-like receptor thermal protein domain associated protein 3; STZ, streptozotocin; NF-κB, nuclear factor κB; LPS, lipopolysaccharide; HFD, high-fat diet; VLDL, very low-density lipoprotein; TLR4, Toll-like receptor 4; RANKL, receptor activator of nuclear factor-κB ligand; miRNAs, microRNAs.
